# Intraurban and Longitudinal Variability of Classical Pollutants in Kraków, Poland, 2000–2010

**DOI:** 10.3390/ijerph120504967

**Published:** 2015-05-06

**Authors:** Hyunok Choi, Steven Melly, John Spengler

**Affiliations:** 1 Department of Environmental Health Sciences, Epidemiology, and Biostatistics, School of Public Health, State University of New York at Albany, One University Place, Rm 153, Rensselaer, NY 12144, USA; 2 Department of Epidemiology and Biostatistics, Drexel University School of Public Health, 3215 Market St., Philadelphia, PA 19104, USA; E-Mail: sjm389@drexel.edu; 3 Exposure, Epidemiology and Risk Program, Department of Environmental Health, Harvard School of Public Health, P.O. Box 15677, Landmark 406 West, 401 Park Drive, Boston, MA 02215, USA; E-Mail: spengler@hsph.harvard.edu

**Keywords:** air pollution, Krakow, coal combustion, exposure misclassification, exposure assessment

## Abstract

In spite of a dramatic decrease in anthropogenic emissions, ambient concentrations of major pollutants have not changed within many urban locations. To clarify the relationship between ambient air quality trend and the population exposures, we compared the intraurban *versus* temporal variability of the collocated measurements of five major air pollutants including particulate matter (PM) with an aerodynamic diameter <10 µm (PM_10_), < 2.5 µm (PM_2.5_), tropospheric ozone (O_3_), sulfur dioxide (SO_2_), and nitrogen dioxide (NO_2_), in Kraków, Poland, during the 2000−2010 period. Strong seasonal trends and overall absence of spatial heterogeneity in PM_10_ and PM_2.5_, except in the traffic monitoring site, were observed across the monitoring network. The range of median PM_2.5_ concentrations during winter (54–64 µg/m^3^) was 3- to 4-times higher than the summer medians (15–26 µg/m^3^) across the sites during 2009−2010. Furthermore, large proportion of PM_10_ appears to be comprised of PM_2.5_ (PM_2.5_/PM_10_ concentration ratios range, 0.5–0.7). At each monitoring site, the Pearson’s correlation coefficients between PM_2.5_ and PM_10_ ranged between 0.944 and 0.963, suggesting a health-relevance of PM_10_ monitoring. One ln-unit increase in PM_10_ was associated with 92%–100% increase in PM_2.5_ concentrations in the same location. While PM_10_ did not demonstrate a clear temporal trend, SO_2_ concentrations steadily declined by 40% during the 2000–2010 period. Summertime median NO_2_ concentration was acutely elevated ‎(70 μg/m^3^ *vs.* 22 μg/m^3^) at the traffic oriented site compared to the city’s central monitoring site. The traffic and the industrial sites were associated with highest number of days during which 24-hour mean PM_10_ and PM_2.5_ concentrations exceeded the European Union standard. Steadily growing contributions by vehicular emissions appear to be associated with the absence of clear trend in PM_10_. Current practices of air quality control within Kraków may not be adequate for the protection of the public’s health.

## 1. Introduction

In spite of reduction in anthropogenic emission of major air pollutants within Europe during the last several decades, such a trend has not been matched by corresponding declines in childhood asthma and allergy prevalence [1,2]. Poland represents an example of such a contradiction. Staring around 1954 under the Communist regime [3], the country has emerged as one of the highest producers and consumers of coal within Europe [4,5,6,7]. For example, total annual emission of particulate matter within Kraków, a city with one of the highest historic levels of air pollution within eastern Europe, is estimated at 150,000 tons during the 1970s [3]. The associated mean ambient PM_10_ concentrations during the same period range between 180 μg/m^3^ (the city center) and 109 μg/m^3^ (the suburbs) [3]. Starting in 1980s, a number of semi-ecologic investigations in Krakow have shown an association between chronic exposures to airborne PM with cause-specific mortality [8,9]. In particular, exposures to particulate matter (PM) have demonstrated robust associations with wide number of health end-points [10,11,12,13,14,15,16,17,18]. At same time, concerns over the deterioration of the natural environment as well as the city’s cultural heritage sites have also grown [19]. Around 1989, Poland’s political transition to democracy following the collapse of communism has led to a substantial decrease in airborne concentrations of SO_2_, black carbon, PM, and airborne heavy metals [20]. Beginning around 1995, both regional and national government bodies have made concerted efforts to improve the air quality [3]. 

To deepen our understanding of the early-life environmental contributions on childhood asthma and neurocognitive impairments, we have been following prospective birth cohort in Krakow since 2000. Our exposure assessment analyses have shown that individual pregnant woman’s personal exposure to particle-bound large PAHs is predominantly influenced by corresponding ambient concentrations [21,22,23]. In addition, there is an extremely high correlation between total sum of eight pro-carcinogenic PAHs and simultaneously monitored PM_2.5_ concentration [10,24,25]. Furthermore, between-person variability in personal exposure to PAHs at given 48-hour window are much smaller than within-person variability [23] or that of the mean ambient concentration [21]. We reported that time-activity pattern of the individual women was not a significant predictor of the personal exposure to particle-bound PAHs [21]. Contrary to our expectation, prenatal exposures to PM_2.5_ and PAHs pose significantly increased risks of intrauterine growth restriction, wheezing symptoms, and asthma during childhood, respectively, in spite of reduction in coal-burning related pollutant emissions [10,11,12,13,14,15,21,22,23,26,27,28,29]. 

Considering the impact of the ambient sources on the personal exposure, the overarching aim of this investigation is to characterize the intraurban trend of five major pollutants across the years 2000−2010. The time-period of our interest corresponds to prenatal and first seven to ten years of the cohort children’s life. This analysis is expected to lay the groundwork for the clarification of the relationship between long-term intraurban trend and chronic exposure profile of each child in the cohort. Furthermore, we posit *a priori* that reduction in coal-burning related emissions is associated with temporally corresponding decline in PM_10_ concentration during the 11-year period of interest. In order to answer this postulate, we: (1) describe the overall trend in the five pollutant concentrations at the six monitors over an 11-year period; (2) compare the size of the season-dependent variability in the five pollutant concentrations according to the site; and (3) explore the influence of the known emission sources and the meteorological factors on PM_10_ and PM_2.5_ concentrations. Pollutants of interest include particulate matter (PM) with aerodynamic diameter < 10 µm (PM_10_), PM < 2.5 µm (PM_2.5_), tropospheric ozone (O_3_), sulfur dioxide (SO_2_), and nitrogen dioxide (NO_2_). 

## 2. Methods

### 2.1. Study Site Characterization

**Figure 1:**
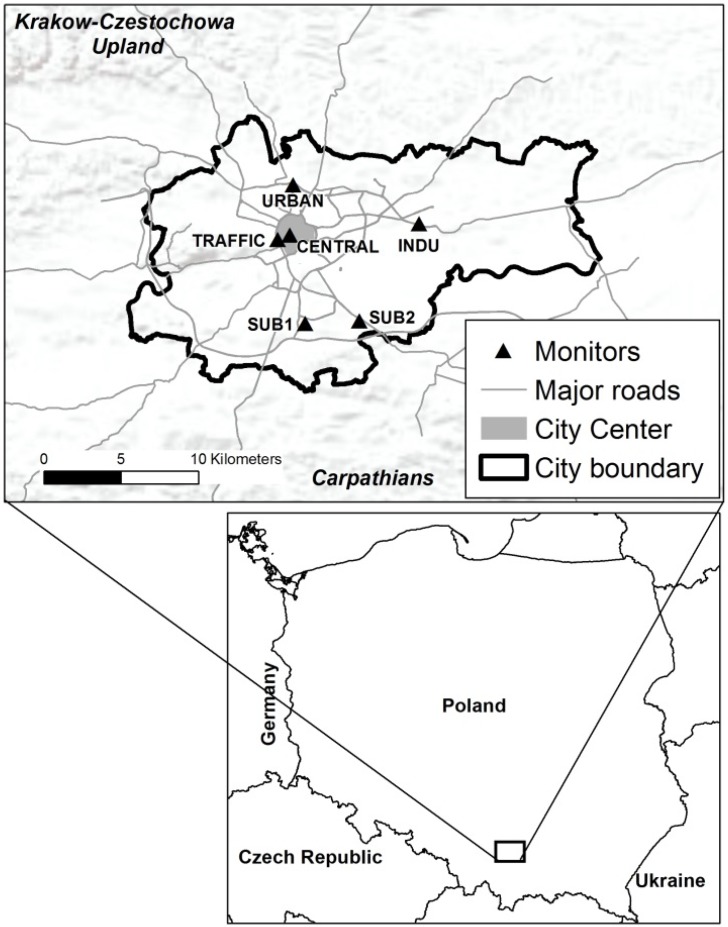
Six monitoring stations cover four districts. The city center is shown in gray. Considering Rynek Główny (CENTRAL) (2000−2004) as the reference point, Krowodrza (URBAN) (2000−2010), Aleja Krasińskiego (TRAFFIC) (2000−2010), Nowa Huta (INDU) (2000−2010), Prokocim (SUB1) (2000-2003), and Kurdwanów (SUB2)(2010) are 3.9 km, 1 km, 9.8 km, 7.5 km, and 7 km away, respectively.

Kraków, Poland, holds a unique position within Polish cultural and academic heritage. Located in southeastern Poland (see Figure 1), it encompasses 327 km^2^ and supports 757,400 inhabitants as of 2005. The city has at least three well-recognized air pollution sources: industrial and coal-fired power plants [20], coal-burning domestic stoves with no or outdated abatement technologies [4,5], and automobile traffic [20]. Following Poland’s annexation to Soviet Union around 1954, a coal-burning steel mill (*i.e*. Lenin Steelworks) and a power plant (*i.e*. Kraków-Łęg plant) were built [3]. To date, these industrial plants continue to provide electricity and heat for new sections of the city. However, collapse of communism in 1989 has reduced the heavy industrial activities within and around the city [3]. Such shift also introduced a gas-operated heating system within the city (covering approximately 30% of the homes) [3]. Accordingly, sulfur dioxide and particulate matter concentrations have steadily decreased. 

Kraków is located in the Vistula river valley surrounded by Carpathian Foothills to the south and The Kraków-Częstochowa Upland to the north (Figure 1). This geographic location has been associated with atmospheric inversions approximately 27% of the entire year, particularly during wintertime [3]. 

### 2.2. The Air Pollutant Sampling and Analysis

The ambient air quality monitoring network in Kraków is operated by Voivodship Inspectorate for Environmental Protection in Krakow (VIEP). Krakow air monitoring network was launched in 1991 in collaboration between the US Environmental Protection Agency and Voivodship Sanitary-Epidemiological Station in Kraków (1968−2001) and the Voivodship Inspectorate for Environmental Protection in Kraków (1992−present) [30]. It has been providing automatic continuous measurement of air pollutants such as SO_2_, NO, NO_2_, NO_x_, CO, O_3_, particulate matter with an aerodynamic diameter less than 10 μm (PM_10_), and from 2009 also PM_2.5_. Laboratory of VIEP got accreditation (contract no AB 176) of Polish Centre for Accreditation (PCA) in 1998 for air quality monitoring testing as a first air monitoring network laboratory in Poland. 

Primary automatic analytic methods for the pollutants during our investigation period include: UV fluorescence for SO_2_ and chemiluminescent method for NO and NO_2_ with gas analyzers produced by Thermo Environmental Instruments Inc, (Franklin, MA, USA, model 43A for SO_2_ and 42 for NO and NO_2_), Environment S.A. (Poissy Cedex, France, model AF 22M for SO2 and AC 32M for NO and NO_2_) and Teledyne Advanced Pollution Instrumentario (San Diego, CA, USA, model API 100A and API 200A). (Thermo Environmental Instruments, Inc Franklin, MA, USA, absorption of UV radiation for O_3_ with ozone analyzers produced by Thermo Environmental Instruments Inc, (Franklin, MA, USA, model 49i) and Environment S.A. (Poissy Cedex, France, model O3 42M); beta attenuation, oscillating microbalance and optical method for PM_10_ and PM_2.5_ with instruments produced by Andersen Instruments, Inc. (Smyrna, GA, USA, model RAAS10), Rupprecht & Patashnick, Co. (Albany, NY, USA, model 1400 TEOM), Met One Instruments, Inc. (Grants Pass, OR, USA, model BAM-1020), Environment S.A. (Poissy Cedex, France, model MP 101M) and GRIMM Aerosol Technik GmbH & Co (Ainring, Germany).

As a laboratory with accreditation (PCA no AB 176) it has implemented system of quality control and assessment in monitoring network according to PN-EN ISO/IEC 17025 norm. It is focused on internal quality control based on qualified staff, instruments calibrations, and completeness of measurements series. To guarantee accuracy and reliability of derived measurements, Krakow VIEP laboratory participate in inter-laboratories comparisons (both in Poland and other EU countries, e.g. Joint Research Center, Ispra, Italy) as well as in national and international proficiency tests. It is a member of AQUILA network. It is also responsible for setting up inter-calibration meeting on the national as well as EU level as a National Calibration Reference Laboratory.

PM_10_, SO_2_, and NO_2_ were monitored in all six stations year round as 24-hr mean concentrations. O_3_ was monitored in four stations (*i.e.*, URBAN, CENTRAL, SUB1, and SUB2) during 2000−2010 period. In contrast, PM_2.5_ were monitored in four stations (*i.e*., URBAN, TRAFFIC, INDU, and SUB2) during 2009−2010 period only. Meteorological data were monitored in INDU, SUB1, and SUB2 sites for temperature and wind speed during January 2000−December 2010. 

Figure 1 shows the approximate location of the six monitoring stations providing data for this analysis. The Rynek Główny (‎CENTRAL) station sits atop a bell tower in Old Town Square of Kraków of approximately 0.04 km^2^ in size. Since construction during 13^th^ century, this largest central plaza in Europe has been a pedestrian square. In contrast, the Aleja Krasińskiego (TRAFFIC) station is located on a busy road in the commercial hub near the CENTRAL‎ site. The Krowodrza (URBAN) station sits on the northern mixed residential and commercial zone as the urban background site. Nowa Huta (INDU) station represents a mixed suburban and industrial zone. Prokocim (SUB1) station represents the newly expanded southern district. Kurdwanów (SUB2), located in southern edge of Krakow, represents urban background site. 

### 2.3. Statistical Analysis

#### 2.3.1. Descriptive Analysis

Present analysis includes the data from four to six monitoring station during January 2000 and December 2010 period for PM_10_, SO_2_, O_3_, and NO_2_ and 2009−2010 period for PM_2.5_. Seasons were defined as summer (June−August), transitional (April, May, September, and October), and winter (November−March). Considering large variability in sample size by site, year and season, extensive non-parametric analyses were conducted for each pollutant. The relevance of predictor variables were examined using Mann–Whitney U-test or the Kruskal-Wallis test depending on the number of categories for the independent variables at α = 0.05 level of significance. There were no pollutant concentrations below the detection limit. All extreme and outlying values were double-checked for accuracy in measurement. Upon positive verification, they were retained in the data. Descriptive analysis was conducted to identify monitoring sites, season, and year, which demonstrate significantly elevated concentrations. 

#### 2.3.2. Linear Regression Model

Pollution variables were natural-log (ln) transformed in order to achieve normal distributions (Komolgorov-Smirnov tests > 0.05) and homoscedasticity. To better understand the relative contribution of PM_10_ on PM_2.5_, a linear regression model of PM_2.5_ was run at four respective monitoring sites which simultaneously monitored PM_10_ on PM_2.5_ in 2009−2010 combined data. The outcome, PM_2.5_, was modeled as a linear function of PM_10_ as the main predictor variable, controlling for temperature, and wind speed, at the four collocated sites. Consistent with earlier investigations, the model regression coefficient was defined as a marker for model accuracy, and adjusted-R^2^ as a marker for model precision [31].

#### 2.3.3. Generalized Linear Mixed Effects Model 

A linear mixed effects model was fit by entering year, month, and sites as indicators variables shown in equation [1] in order to detect a trend without imposing a structure on the relationship. The reference categories were set as Saturday, December, CENTRAL and Year 2010 for the variables, weekday, month, site, and year, respectively:

(1) $$\begin{array}{l} \ln{\left(X\right)}_{i}=\alpha +\sum_{n=1}^{10}\beta_{n}\;{\left(Year\right)}_{i}+\sum_{m=1}^{11}\gamma_{m}{\left(Month\right)}_{i}+\sum_{o=1}^{6}\theta_{o}{\left(Weekday\right)}_{i}+\sum_{p=1}^{5}\delta {\left(Site\right)}_{i}+\;\zeta (Temp)\;+\;\eta (WS) \end{array}$$

Where *α* represent the y-intercept; *β_n_, γ_m_, θ_o_, δ_p_, ζ*, and *η*, respectively, represent the slope of the independent variables. All independent variables were forward selected if the probability of given variable in the model showed F ≤ 0.05, and removed it if the probability of the model had F ≥ 0.10. 

The pollutant, X, concentration was predicted by rewriting equation [1] as follows:

(2) $$[X]\;=\;EXP[\alpha ]\;\times \;EXP[\sum_{n=1}^{10}\beta_{n}\;{\left(Year\right)}_{i}+\sum_{m=1}^{11}\gamma_{m}{\left(Month\right)}_{i}+\sum_{o=1}^{6}\theta_{o}{\left(Weekday\right)}_{i}+\sum_{p=1}^{5}\delta {\left(Site\right)}_{i}+\;\zeta (Temp)\;+\;\eta (WS)].$$

In order to compare the relative importance of each predictor (e.g., year), we calculated concentration impact factor of given predictor variable as *IF_i_* = exp[∑β*_i_* × (variable)*_i_*], holding all other variables constant [32]. That is, the impact factor refers to concentration change associated with a given predictor variable apart from the baseline level (*i.e*. y-intercept). Accordingly, the intercept term of the equation [2] reflects the mean concentration at the baseline level for all predictors (e.g., Saturday, July, CENTRAL site, Year 2010, ‎ wind speed decrease by ≥ 1 m/s, and one °C reduction in ambient temperature from 14.60 °C). The impact factor at the reference level for a given variable equals 1, given that exp[0] = 1. Accordingly, impact factor > 1 indicates predicted concentration, which is greater than the baseline concentration. On the other hand, impact factor < 1 indicates a lower predicted concentration for a given predictor variable relative to the baseline level. We conducted all statistical analyses in SAS version 9.3 (SAS Institute Inc., Cary, NC, USA). All figures were generated using IBM^®^SPSS^©^ version 22.0 (SPSS Inc., Chicago, IL, USA).

## 3. Results and Discussion

### 3.1. Descriptive Analyses

#### 3.1.1. PM_10_

**Figure 2:**
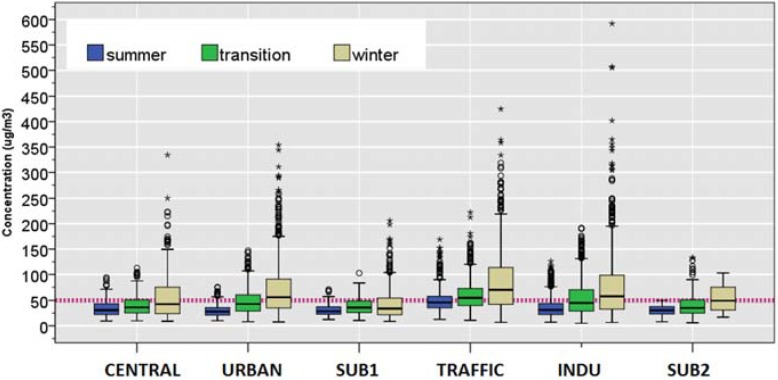
Distributions of daily concentrations of PM_10_ during summer (June, July, and August); transition (April, May, September, and October); winter (November through March). The dotted line shows the EU standard of 50 µg/m^3^ for PM_10_. Boxes show 25th, 50th and 75th percentile; the whiskers show 5th and the 95th percentile values. The symbols, ○ and *, represent measurements that are >1.5- and >3-fold of the interquartile range.

Table 1 and Figure 2 show site-specific central tendencies and the exceedance days (>25 μg/m^3^ for PM_2.5_ and >50 μg/m^3^ for PM_10,_ based on the current EU standard), and PM_2.5_/PM_10_ ratios. During summer, TRAFFIC (21%) and INDU (16%) sites were respectively associated with the highest number of days during which 24-hour mean PM_10_ concentration exceeded 50 µg/m^3^ (the current EU standard) compared to the CENTRAL site (3%). Two suburban sites (SUB1 and SUB2) were associated with even fewer number of exceedance days during the transition season (5 and 2%, respectively). Similar trend was seen in the number of exceedance days for PM_10_ during the transition season for TRAFFIC (31%) and INDU (37%) site, compared to the CENTRAL site (9%). Such spatial variability was particularly acute during winter, in which TRAFFIC and INDU had highest proportion of exceedance days (39% and 51%, respectively, *vs.* 13% in CENTRAL site). INDU was associated with a widest range for daily PM_10_ concentration (6.6−592 μg/m^3^) during winter (Figure 2). Due to the high mean summer PM_10_ concentration at TRAFFIC site, the mean winter/summer ratio for PM_10_ concentrations were lower for TRAFFIC (1.5), compared to the INDU (1.9) as well as URBAN (2.0). 

Examining the city-wide average PM_10_ annual levels of the 11-year period, we observe year to year variability but little evidence regarding improvement of PM_10_ air quality (43.7 ± 26.5; 35.9 ± 24.4; 68.1 ± 46.5; 60.5 ± 45.4; 58.7 ± 39.6; 57.4 ± 44.6; 70.6 ± 64.3; 57.4 ± 42.4; 54.8 ± 39.3; 64.0 ± 40.9; and 57.1 ± 39.5 μg/m^3^). Such annual mean is considerably higher than those reported in other urban background sites in European countries during the 1998−2002 period (Germany 28–38 μg/m^3^; Spain 31−42 μg/m^3^; Sweden 17−23 μg/m^3^; the Netherlands 25 μg/m^3^; United Kingdom 25 μg/m^3^; Switzerland 24 μg/m^3^) [33]. 

#### 3.1.2. PM_2.5_

**Figure 3:**
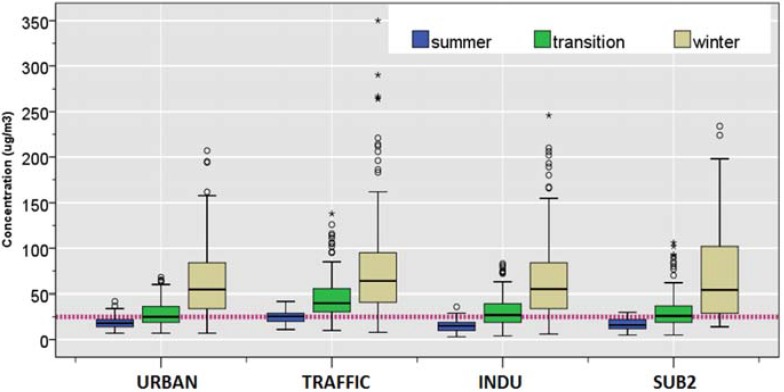
Distributions of daily concentrations of PM_2.5_ during summer (June, July, and August); transition (April, May, September, and October); winter (November through March) of PM_2.5_, The dotted line shows the EU standard of 25 µg/m^3^.

As indicated by Table 1 and Figure 3, site-specific daily mean PM_2.5_ concentrations showed a distinct seasonal trend. The median PM_2.5_ concentration during summer were highest at the TRAFFIC site (26 μg/m^3^) and uniform overall at other sites (18 μg/m^3^ for URBAN; 15 μg/m^3^ for INDU; and 16 μg/m^3^ for SUB2). In addition, the same site was also associated with higher number of exceedance days (6%) as well as highest mean PM_2.5_ concentration (25.2 ± 7.0 μg/m^3^), compared to all other sites during the summer. 

During the transition season, TRAFFIC site was associated with the highest median (40 μg/m^3^) as well as a highest number of exceedance days (8%), whereas other sites demonstrated overall uniform median concentration (25 μg/m^3^ at URBAN; 27 μg/m^3^ at INDU; 26 μg/m^3^ at SUB2). In contrast, similar numbers of exceedance days as well as the mean were observed during winter for the URBAN, TRAFFIC, and INDU sites (11%, 12%, and 13%, respectively) compared to the SUB2 site. Accordingly, such seasonal pattern was associated with highest median winter/summer ratios for the INDU and SUB2 (3.7 and 3.4, respectively) and the lowest winter/summer ratio for the TRAFFIC (2.5). 

Taking all four sites together, the combined annual mean concentrations of PM_2.5_ were 43.6 ± 31.6 μg/m^3^ during 2009 and 46.7 ± 43.1 μg/m^3^ during 2010. Such concentrations far exceeded the annual mean EU standard of 10 μg/m^3^ [34]. 

### 3.2. PM_2.5_ and PM_10_ Relationship

#### 3.2.1. PM_2.5_/PM_10_ Concentration Ratio 

The relative abundance of fine fraction to PM_10_ is shown according to site and season in Table 1. The PM_2.5_/PM_10_ ratio exhibited a distinct seasonality. However, there were no clear differences across the sites in the ratios. During the summer, the mean ratio ranged between 0.5 and 0.7 among the four sites. During the winter, the same ratio ranged between 0.7 and 0.8. Overall, these ratios are consistent with ratios observed in other urban and semi-urban locations within Europe, including Netherlands, Germany, and Spain at 0.8 [33]. However, considerably lower ratios have been observed more frequently in U.S. locations (range, 0.3–0.7) [35], in Sweden and in the Canary Islands (0.4), Santiago, Chile (range, 0.4−0.6) [32], or Athens, Greece (range, 0.4−0.6) [32]. 

#### 3.2.2. Pearson’s Correlation Coefficients 

The ln-transformed PM_2.5_ and PM_10_ across the sites were associated with coefficients between 0.826 and 0.963 based on the collocated measurements on given day (Table 2). In particular, at each site (*i.e*., URBAN, TRAFFIC, INDU, and SUB2, respectively), the correlations between PM_2.5_ and PM_10_ ranged between 0.944 and 0.963. 

Within URBAN, INDU, and SUB2 sites, one ln-unit increase in PM_10_ was able to explain 91%, 93%, and 91% of total variability in PM_2.5_, respectively (Table 3). In addition, one ln-unit increase in PM_10_ concentration predicted 92%, 100%, and 99% increase in PM_2.5_ in the same sites. In contrast, the same model for the TRAFFIC site was associated with lower accuracy (*β* = 0.74) and precision (adjusted R^2^ = 0.60) in predicting PM_2.5_ concentration per same unit increase in PM_10_.

#### 3.2.3. SO_2_

**Figure 4:**
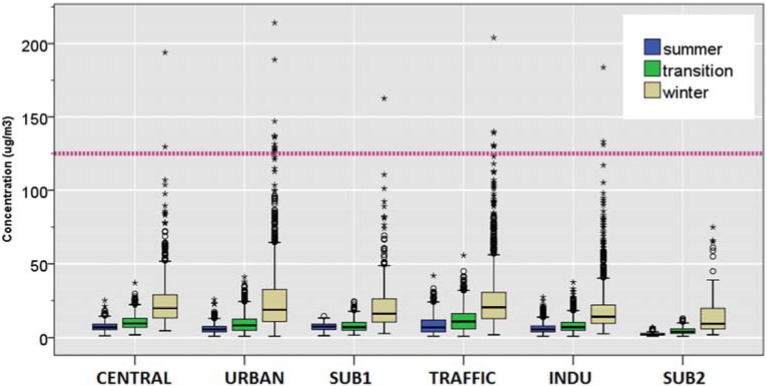
Distributions of daily concentrations SO_2_ by season. The dotted line shows the EU standard of 125 µg/m^3^ for SO_2_.

Clear, yet, overall consistent seasonal variability in the median SO_2_ concentration was observed across the sites in the 2000−2010 combined data (Figure 4). As shown in Table 4, the mean winter SO_2_ concentration was approximately 3-times higher than that during the summer, except in SUB2. Spatial variability in SO_2_ concentration was examined by standardizing the concentration at given site by that at the CENTRAL site, collocated measurement on given date (Table 4). During summer, SO_2_ concentration was highest at the TRAFFIC site relative to the CENTRAL site (1.8 *vs.* 1.1 in all remaining sites). A same pattern for SO_2_ was again observed during the transition season with highest ratio for the TRAFFIC site (1.8) compared to the remaining sites (range, 0.8−1.2). However, during the winter, little differences were observed among the URBAN, TRAFFIC, INDU, and SUB1 sites (range, 0.9−1.3). 

During the years 1968−1973, mean daily SO_2_ concentration in Krakow was 119 µg/m^3^ with various sub-sections of the city reporting even higher mean annual concentration of SO_2_ (80−120 µg/m^3^ ) [3]. In contrast, the median concentration during the winter over the years 2000−2010 period (Table 4 and Figure 4) at the CENTRAL (20 µg/m^3^) and the INDU (14 µg/m^3^) sites reflect a reduction in SO_2_ contribution to ambient air pollution in Kraków. 

**Figure 5:**
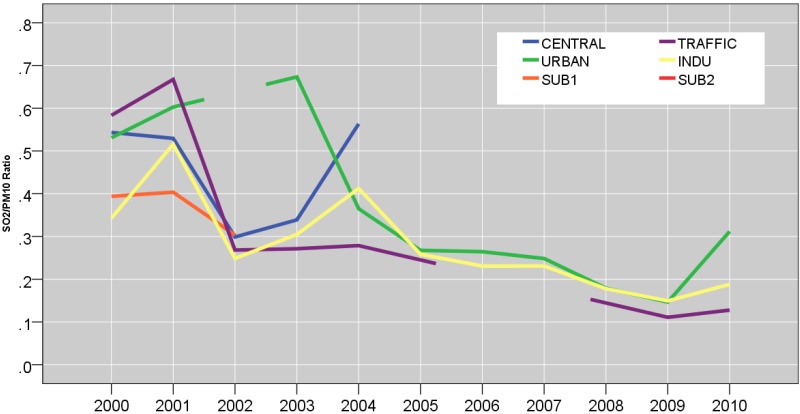
SO_2_/PM_10_ concentration ratio according to site and year.

Spatiotemporal variability in relative abundance of SO_2_ against PM_10_ was compared as annual mean SO_2_/PM_10_ ratio based on collocated 24-hour measurements (Figure 5). Wide variability in SO_2_/PM_10_ ratio was observed between the sites at the onset of the study period. The three sites in the northeastern portion of the city (CENTRAL, URBAN, and TRAFFIC) are associated with the highest ratios (range, 0.5−0.6), while SUB1 and INDU site have ratios < 0.4. However, the rates at all sites, except URBAN, were associated with a uniform decline to 0.3 in 2002. Subsequently, the annual mean SO_2_/PM_10_ ratios decreased steadily in URBAN, INDU, and TRAFFIC sites between 2004 and 2009. 

**Table 1:** Concentration distributions for PM_10_ (years 2000−2010) and PM_2.5_ (years 2009–2010). ^a^ refers to the number of days that exceeded the current EU standard.

	PM_2.5_ (μg/m^3^)					PM_10_ (μg/m^3^)					PM_2.5_/PM_10_
	N	Mean ± SD	Min	Max	>25 ^a^ (%)	N	Mean ± SD	Min	Max	>50 ^a^ (%)	Mean ± SD
CENTRAL											
summer						305	33.7 ± 14.6	9.4	93.9	33(3%)	
transition						459	39.8 ± 18.8	9.8	112.6	123 (9%)	
winter						507	55.3 ± 43.0	9.2	334.3	210 (13%)	
winter/summer							1.4				
URBAN											
summer	90	18.7 ± 6.5	7.0	42.0	12 (1%)	495	29.2 ± 11.0	10.0	75.0	18 (2%)	0.7 ± 0.1
transition	120	28.8 ± 13.7	7.0	68.0	57 (4%)	751	47.5 ± 24.3	8.0	147.0	281 (21%)	0.6 ± 0.1
winter	202	62.8 ± 37.0	7.0	207.0	180 (11%)	1069	69.5 ± 49.6	7.7	354.0	598 (36%)	0.8 ± 0.1
winter/summer		3.0					2.0				
TRAFFIC											
summer	116	25.2 ± 7.0	11.0	42.0	58 (6%)	548	49.1 ± 22.8	12.8	169.1	214 (21%)	0.6 ± 0.1
transition	132	46.1 ± 24.8	10.0	138.0	113 (8%)	700	59.2 ± 29.4	11.0	222.4	414 (31%)	0.7 ± 0.1
winter	209	76.6 ± 52.6	8.0	350.0	191 (12%)	947	85.7 ± 58.9	6.8	424.8	644 (39%)	0.7 ± 0.1
winter/summer		2.5					1.5				
INDU											
summer	109	14.9 ± 6.6	3.0	36.0	7 (1%)	890	35.8 ± 18.7	7.0	126.0	158 (16%)	0.5 ± 0.1
transition	167	30.9 ± 16.3	4.0	83.0	90 (7%)	1147	53.0 ± 32.1	5.0	191.0	502 (37%)	0.6 ± 0.1
winter	242	65.4 ± 42.1	6.0	246.0	211 (13%)	1491	73.2 ± 57.1	6.6	592.0	856 (51%)	0.8 ± 0.1
winter/summer		3.7					1.9				
SUB1											
summer						202	31.4 ± 11.5	12.4	70.5	14 (1%)	
transition						297	38.6 ± 16.8	10.5	102.6	72 (5%)	
winter						385	44.8 ± 33.0	8.6	206.0	110 (7%)	
winter/summer							1.2				
SUB2											
summer	89	16.8 ± 6.3	5.0	30.0	9 (1%)	84	30.2 ± 10.0	8.0	50.0	0 (0%)	0.6 ± 0.1
transition	118	31.6 ± 21.1	5.0	106.0	60 (5%)	119	42.4 ± 26.8	6.0	133.0	30 (2%)	0.7 ± 0.1
winter	61	71.8 ± 56.6	14.0	234.0	49 (3%)	30	55.0 ± 27.1	17.0	103.0	14 (1%)	0.8 ± 0.1
winter/summer		3.4					1.6				

**Table 2:** Pearson’s correlation coefficients between PM_2.5_ and PM_10_ among the sites . ** denotes correlation coefficient which are significant at a < 0.01.

		PM_10_						PM_2.5_			
		CENTRAL	URBAN	SUB1	TRAFFIC	INDU	SUB2	URBAN	TRAFFIC	INDU	SUB2
**PM_10_**	CENTRAL	1	0.829 **	0.875 **	0.835 **	0.833 **					
	URBAN		1	0.841 **	0.751 **	0.898 **		0.961 **	0.951 **	0.928 **	
	SUB1			1	0.737 **	0.825 **					
	TRAFFIC				1	0.836 **	0.903 **	0.880 **	0.944 **	0.911 **	0.904 **
	INDU					1	0.888 **	0.886 **	0.918 **	0.963 **	0.896 **
	SUB2						1		0.871 **	0.826 **	0.947 **
**PM_2.5_**	URBAN							1	0.967 **	0.957 **	
	TRAFFIC								1	0.951 **	0.953 **
	INDU									1	0.947 **
	SUB2										1

**Table 3:** Site-specific model of PM_2.5_ (outcome) as a linear function of PM_10_ (predictor), adjusting for temperature and wind speed.

Site Name	Predictor	β	(95% CI)	Adjusted-R^2^
URBAN	y-intercept	−0.14	(−0.32 0.05)	
	(Ln) PM_10_	0.92	(0.87 0.97)	0.914
TRAFFIC	y-intercept	0.62	(−0.31 1.54)	
	(Ln) PM_10_	0.74	(0.51 0.97)	0.602
INDU	y-intercept	−0.53	(−0.65 −0.40)	
	(Ln) PM_10_	1.00	(0.97 1.03)	0.931
SUB2	y-intercept	−0.38	(−0.57 −0.18)	
	(Ln) PM_10_	0.99	(0.94 1.04)	0.909

**Table 4:** Concentration distributions for SO_2_, O_3_, and NO_2_ by site and season, 2000−2010.

	SO_2_				O_3_				NO_2_			
	N	Mean ± SD	MIN	MAX	N	Mean ± SD	MIN	MAX	N	Mean ± SD	MIN	MAX
CENTRAL												
summer	360	7.7 ± 3.4	1.3	25.1	29	38.0 ± 12.8	23.5	72.0	246	23.6 ± 6.7	9.7	43.5
transition	545	10.6 ± 5.1	1.9	37.0					399	29.2 ± 9.4	8.9	60.1
winter	672	24.2 ± 17.1	4.7	193.9					621	35.2 ± 13.1	11.3	93.6
winter/summer		2.8								1.5		
URBAN												
summer	711	6.1 ± 3.3	1.0	25.8	649	48.3 ± 16.7	14.0	130.6	611	29.7 ± 8.7	8.5	59.0
transition	1051	9.7 ± 6.2	1.0	41.1	872	33.8 ± 16.6	3.0	89.6	981	33.9 ± 10.7	8.6	68.5
winter	1397	25.3 ± 21.9	1.0	214.1	1131	24.5 ± 16.0	2.0	85.2	1192	37.7 ± 16.5	7.0	130.0
winter/summer		3.3				0.5				1.2		
TRAFFIC												
summer	915	8.6 ± 5.9	1.0	41.9					874	70.5 ± 15.5	25.7	125.7
transition	1236	12.0 ± 7.7	1.0	55.8					1226	69.0 ± 16.4	21.7	123.5
winter	1582	25.0 ± 19.2	2.0	204.1					1526	62.3 ± 19.3	20.8	152.6
winter/summer		2.9								0.9		
INDU												
summer	849	6.5 ± 3.7	1.0	27.3					884	25.0 ± 7.1	7.0	53.9
transition	1116	8.3 ± 4.8	1.0	37.5					1152	28.7 ± 9.1	2.7	61.0
winter	1509	18.3 ± 14.9	2.7	183.7					1586	35.2 ± 14.3	7.0	130.0
winter/summer		2.5								1.3		
SUB1												
summer	182	7.6 ± 2.7	1.3	14.7	179	49.3 ± 13.9	15.9	109.4	169	25.1 ± 7.9	7.3	49.7
transition	266	8.2 ± 4.4	1.7	24.4	204	41.2 ± 17.4	5.7	78.8	261	28.8 ± 9.2	6.7	57.1
winter	350	21.6 ± 18.1	2.8	162.6	287	30.6 ± 15.9	5.2	73.6	302	32.6 ± 13.2	6.7	80.8
winter/summer		2.2				0.6				1.3		
SUB2												
summer	87	2.7 ± 1.4	1.0	7.0	86	44.5 ± 14.1	18.0	78.0	86	31.5 ± 9.2	14.0	56.0
transition	120	4.8 ± 2.9	1.0	13.0	117	32.5 ± 14.7	4.0	70.0	112	31.3 ± 11.2	12.0	68.0
winter	58	17.6 ± 18.2	2.0	75.0	69	21.7 ± 19.0	1.0	62.0	69	40.2 ± 16.0	17.0	87.0
winter/summer		4.8				0.3				1.3		

#### 3.2.4. NO_2_

**Figure 6:**
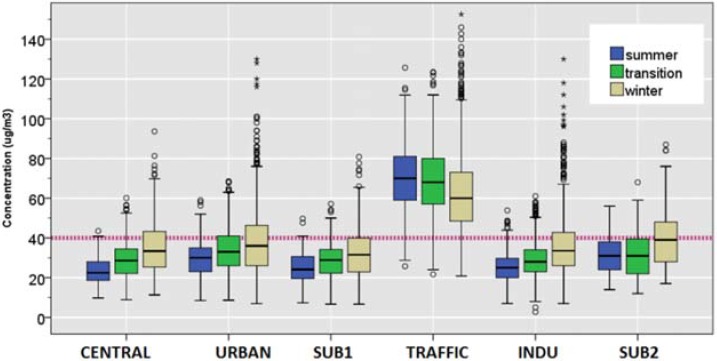
Distributions of daily NO_2_ concentration (µg/m^3^) by season. The dotted line shows the EU annual mean standard of 40 µg/m^3^.

**Figure 7:**
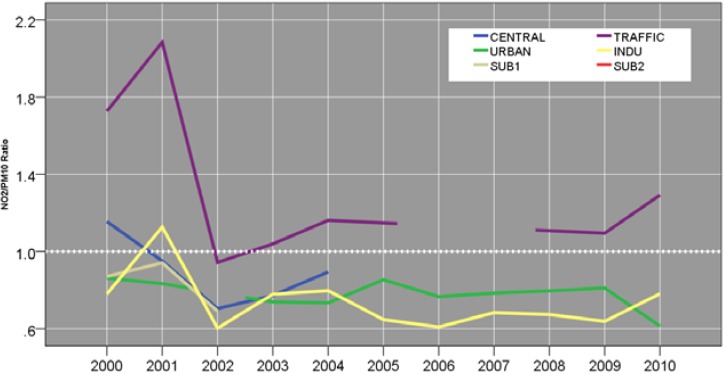
NO_2_/PM_10_ ratio by site and year.

In contrast to other pollutants, NO_2_ exhibited larger spatial heterogeneity in their median concentrations. Such heterogeneity was particularly apparent during summer (Table 4 and Table 5, Figure 6 and Figure 7). During the summer, the median NO_2_ at the TRAFFIC site was 3-times higher than that at the CENTRAL ‎site (70 *vs.* 22 μg/m^3^). During all seasons, the median NO_2_ was lowest at the CENTRAL ‎site compared to all other sites. This reflects the fact that the CENTRAL station sits within a square, protected from automobile traffic. In contrast, TRAFFIC was also the site in which the inverse trend was observed against the season. While all other sites were associated with an elevated median NO_2_ concentration during winter, the median NO_2_ concentration was highest during summer at the TRAFFIC site. 

As shown in Figure 6, exceedance of the EU annual standard of 40 μg/m^3^ was observed most frequently at the TRAFFIC site. Largest seasonal fluctuation for the median NO_2_ was observed at CENTRAL (Winter/summer = 1.5). During the winter, the median NO_2_ concentration in TRAFFIC was approximately 1.8-times higher than that at the CENTRAL ‎(60 μg/m^3^ *vs.* 33 μg/m^3^) (Table 4). 

Relative abundance of NO_2_ against PM_10_ was estimated as annual mean NO_2_/PM_10_ ratio based on collocated 24-hour measurements (Figure 6). TRAFFIC was the only site for which NO_2_/PM_10_ ratio consistently remained greater than unity.

Furthermore, Table 5 shows the spatial variability in concentration ratios of PM_10_/PM_10,_ considering the CENTRAL site in the denominator. Overall, there was little difference in PM_10_ concentration at URBAN, INDU and SUB1 sites, considering the PM_10_ concentration CENTRAL site as the reference. Regardless of season, the ratios of PM_10_ concentrations of given site, relative to CENTRAL site did not markedly differ from unity (range, 0.9−1.3). On the other hand, the median PM_10_ concentration at the TRAFFIC site was 50% (during summer and transition season) and 60% higher (during winter) than those at the CENTRAL site (range, 1.5−1.6). As shown in Table 5, spatial concentration ratios were highest at TRAFFIC for PM_10_, SO_2_, and NO_2_ regardless of season. 

**Table 5:** Spatial concentration variability ratios using collocated monitors in 2000−2010 combined data. Denominator (reference) is set as the concentration of given pollutant at CENTRAL.

		Numerator		URBAN		TRAFFIC		INDU		SUB1
	Denominator		CENTRAL		CENTRAL		CENTRAL		CENTRAL	
			N	Mean ± SD	N	Mean ± SD	N	Mean ± SD	N	Mean ± SD
**PM_10_**	Summer		70	1.2 ± 0.4	279	1.5 ± 0.5	263	1.1 ± 0.4	161	1.2 ± 0.3
	Transition		152	1.3 ± 0.4	419	1.5 ± 0.5	420	1.3 ± 0.6	271	1.1 ± 0.3
	Winter		135	1.3 ± 0.4	496	1.9 ± 0.7	483	1.3 ± 0.4	263	1.2 ± 0.4
	Overall		357	1.3 ± 0.4	1194	1.7 ± 0.6	1166	1.3 ± 0.5	695	1.1 ± 0.3
**SO_2_**	Summer		253	1.1 ± 0.5	321	1.8 ± 0.6	281	1.1 ± 0.5	132	1.1 ± 0.4
	Transition		464	1.2 ± 0.5	522	1.8 ± 0.6	411	0.9 ± 0.4	265	0.8 ± 0.5
	Winter		604	1.3 ± 0.4	650	1.3 ± 0.3	575	0.9 ± 0.3	333	0.9 ± 0.3
	Overall		1321	1.2 ± 0.4	1493	1.6 ± 0.6	1267	0.9 ± 0.4	730	0.9 ± 0.4
**NO_2_**	Summer		92	1.3 ± 0.3	197	3.3 ± 0.7	213	1.2 ± 0.3	128	1.1 ± 0.2
	Transition		262	1.1 ± 0.3	376	2.5 ± 0.7	297	1.1 ± 0.3	200	1.0 ± 0.2
	Winter		347	1.1 ± 0.3	584	1.8 ± 0.4	580	1.0 ± 0.2	269	1.0 ± 0.3
	Overall		701	1.1 ± 0.3	1157	2.3 ± 0.8	1090	1.0 ± 0.2	597	1.0 ± 0.3

#### 3.2.5. O_3_

**Figure 8:**
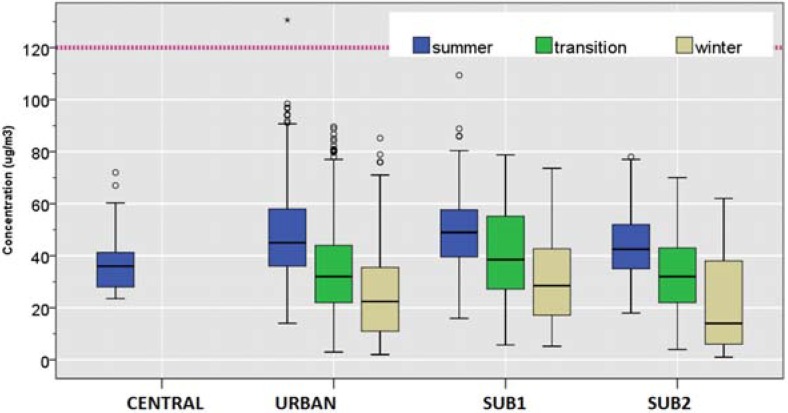
Distributions of daily mean tropospheric ozone concentration by season. The dotted line on (a) shows the EU standard of 120 µg/m^3^ for maximum daily 8-hour mean.

Compared to the summer O_3_ concentration, the median level during transition and winter were 20% and 50%, respectively, of the summer level at the URBAN station. In SUB1 station, the median O_3_ decreased by 20% during transition, and by 40% during winter compared to the median during summer. Within SUB2 station, the median O_3_ decreased by 26% during the transition season, and by 67% during winter compared to the median during summer (Table 4 and Figure 8). Such levels remained well under the EU standard, 120 µg/m^3^, based on the daily 8-hour mean. 

### 3.3. Regression Model Results 

**Figure 9:**
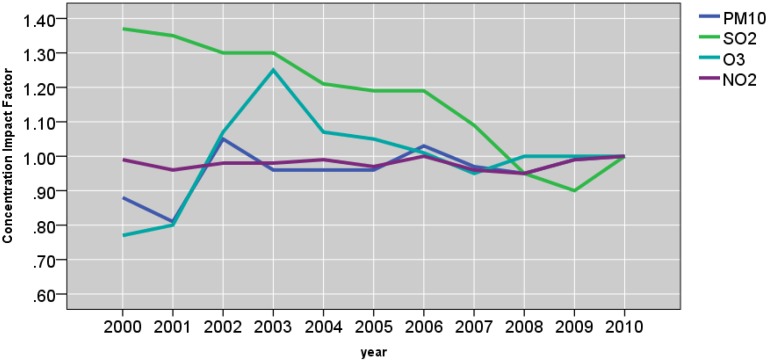
Yearly concentration impact factors.

**Figure 10:**
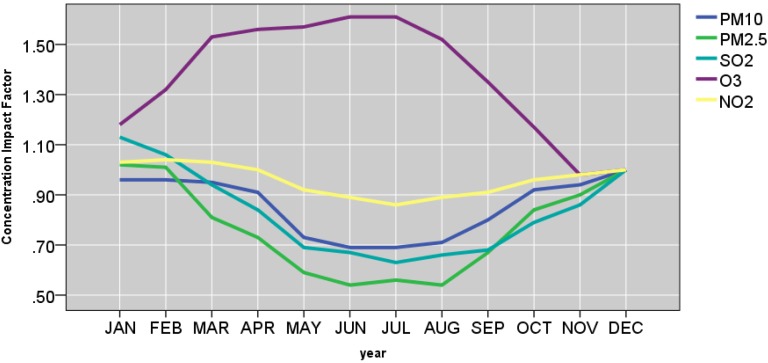
Monthly concentration impact factors.

**Figure 11:**
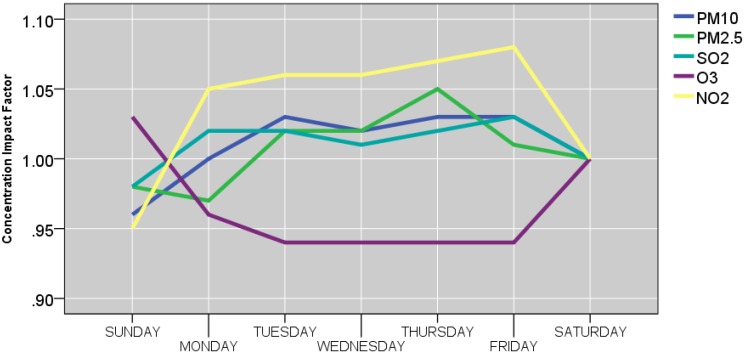
Weekday concentration impact factors.

Figure 9 , Figure 10 and Figure 11 and Table A1 show the effects of the site, year, month, season, day of the week, temperature, and wind speed on the pollutants. The mean predicted concentration of PM_10_, PM_2.5_, SO_2_, NO_2_, and O_3_ were 5.38 μg/m^3^, 5.61 μg/m^3^, 2.55 μg/m^3^, 4.31 μg/m^3^, and 3.19 μg/m^3^, respectively at the reference points (*i.e*., site CENTRAL‎, year 2010, summer, Saturday, wind speed ≥ 0.90 m/s, and temperature ≥ 14.60 °C). 

#### 3.3.1. Site Effect

Mean concentration difference at each site relative to CENTRAL‎ is shown for the pollutants based on regression coefficients and standard error in Table A1. The mean concentrations of PM_10_ were 19% higher in TRAFFIC and 10% higher at INDU site, compared to the CENTRAL site. For PM_2.5_, CENTRAL, SUB1, and SUB2 represent the reference sites due to missing measurements in these sites. For PM_2.5_, the mean concentrations at URBAN, TRAFFIC, and INDU sites ‎were not markedly different from the reference sites (impact points range, 1.00−1.14). Similarly, mean concentration at TRAFFIC was 13% higher than that at CENTRAL‎, while the SUB1 and SUB2 sites had mean SO_2_ were 9% and 7% lower than that at CENTRAL‎. Holding all other variables constant, the mean concentration of NO_2_ was 42% higher than that in CENTRAL‎. 

#### 3.3.2. Year Effect

As shown in Figure 9, yearly trend of PM_10_ and NO_2_ remained relatively constant over 2000−2010 period. The results of regression models (Table A1) and the concentration impact factor show that the mean concentrations of PM_10_ and NO_2_ remained overall constant throughout the monitoring period. Specifically, concentration impact factors for PM_10_ ranged between 0.88 and 1.03 over the period 2000–2010 or, differed from the reference point by 1% per year. The yearly effect of 2009 on PM_2.5_ shows that there was a 3% increase in mean concentration, after accounting for other variables, including temperature and wind speed. For NO_2_, the concentration impact factors remained near 0.99 throughout the monitoring period. In contrast, there was a dramatic decrease in annual mean SO_2_ concentration over the same period (Figure 8). Considering year 2010 as the reference point (impact point, 1), the impact factor of SO_2_ steadily decreased from 1.37 in 2000 to 0.90 in year 2009. The yearly trend of O_3_ was 20%−23% lower than the reference year 2010. However, the impact factor increased by 7%−25% in year 2002−2003 period. Subsequently, it leveled off towards unity in subsequent years. 

Such PM_10_ observation is consistent with a more recent analysis by Junninen *et al.* (2009), which has not shown a clear long-term PM trend in peak ambient levels for PM since our investigation during 2000−2002 period [4]. For example, during the winter of 2005, the peak ambient concentrations for PM with an aerodynamic diameter <10 µm (PM_10_) was 400 μg/m^3^ and peak ambient benzo[*a*]pyrene was 200 μg/m^3^ [4]. 

#### 3.3.3. Month Effect

As shown in Figure 10, the effect of the month is strongly correlated with season for all pollutants of our interest. Considering December as the reference point (impact factor, 1) monthly concentration impact factors reach their lowest points during the May, June, July and August for PM_2.5_, PM_10_, SO_2_, and NO_2_. Specifically, July was associated with 14% decrease in mean NO_2_. When the temperature and wind speed variables were excluded from the regression models, the month of July was associated with 7% decrease in mean NO_2_. For O_3_, the effect of the month was in opposite direction. Between January and May period, the impact factor steadily increased from 1.18 to 1.57. Suring summer, the impact factor peaked between 1.52 and 1.61. It subsequently subsided from 1.35 to 0.98 during September to November period. 

#### 3.3.4. Weekday Effect

As shown in Figure 11, weekday played most visible role in NO_2_ and O_3_ concentrations, but not in PM_2.5_ and PM_10_ concentrations. During weekdays (Monday−Friday), the mean concentration of NO_2_ increased approximately 6% compared to the reference day (Saturday). For O_3_, the same period was associated with impact factor decrease by 6% compared to the reference (Saturday). 

Contrary to our expectation, our analysis demonstrates overall poor ambient air quality in Kraków, with little improvement during the 11-year period. Such a pattern reflects the complex interplay of the sources, valley setting, and meteorological factors [4]. Clear seasonal trends of PM_10_, PM_2.5_, SO_2_, and NO_2_ suggest the importance of the both coal-burning as well as traffic sources. The levels of PM_10_ and PM_2.5_ seen in this study reflect vast improvement in air quality of Kraków, compared to that during the Communist regime. For example, average annual concentration of PM_10_ changed from 154 µg/m^3^ in 1993 to 49 µg/m^3^ in 2007 [36]. During 1992–1999 period, ambient concentration of lead ranged between 0.006 and 0.434 μg/m^3^ (near residential area); 0.016–0.739 μg/m^3^ (near the industrial area); and 0.021–1.147 μg/m^3^ near roads [20]. Recent analysis estimated >50% of PM_10_ in Kraków are contributed by coal burning for residential heating, and rest to automobile traffic and industrial power plants [4]. As recently as 2005, 24-hour mean concentration of airborne benzo[a]pyrene (B[a]P) at 200 ng/m^3^ has been observed during winter [4]. Small domestic stoves/boilers for heating represent the primary contributors of airborne PM and polycyclic aromatics hydrocarbons during winter [4,5]. Krakow also receives air pollution from the Upper Silesia coal region [37]. 

However, the ambient levels of PAH and heavy metals continue to be high in the central section of the city, because of increasing traffic (especially diesel) and continued coal burning by industrial and residential sources [4]. In addition, transition to a market economy expanded the vehicular fleet in Kraków. Traffic density in the city center is estimated at 2500–3000 cars/hour between 7 am to 5 pm, and subsequently decreases to 200–500 cars/hour during the night [38]. In residential area, mean traffic is estimated at 50 cars/hour [38]. 

Our analysis suggests that NO_2_ represents a dominant species in TRAFFIC site. TRAFFIC was the only site for which NO_2_/PM_10_ ratio consistently remained greater than unity. In addition, the spatial concentration variability ratios (PM_10_/PM_10_, SO_2_/SO_2_, and NO_2_/NO_2_, considering CENTRAL concentration as the denominator) suggest that NO2 and other vehicular emission factors are considerably higher only at the TRAFFIC site. 

Three sites in northwestern portion of the city (CENTRAL, URBAN, and TRAFFIC) are associated with the highest SO_2_/PM_10_ ratios (range, 0.5−0.6), while SUB1 and INDU site have SO_2_/PM_10_ ratios < 0.4. While CENTRAL, URBAN, and TRAFFIC in northwestern portion of the city comprises high pollution and southeastern section of (comprised of INDU, SUB1, and SUB2) had overall lower pollution level and higher seasonal fluctuation in all of the pollutants. Our present observation is consistent with growing emissions from the mobile sources [20] as well as rising secondary particle formation since 1989 [4]. 

While domestic coal-burning boilers and local heating facilities without an abatement strategy have been replaced by gas-burning boilers [3], it remains unclear how effective they are as remediation strategy. Overall steady reduction in coal-burning related emission has failed to produce corresponding decrease in a number of childhood morbidity outcomes [3,36]. Furthermore, the average prevalence of childhood asthma has increased by 9% during 1993−2003 period in Poland [36]. More effective strategies for air quality improvement are needed for the protection of the health of the population in Krakow. 

## 4. Conclusions

Air quality in Krakow did not improve during the 2000−2010 period. Such a pattern is observed in spite of a dramatic decline in ambient SO_2_ concentrations over the 11-year period. The site-combined annual mean PM_10_ remained overall constant and considerably higher than the annual value reported for other urban background levels in other European cities. Both PM_10_ and PM_2.5_ exhibited clear season-dependent and site-specific variability in their mean concentration. Specifically, PM_10_ and PM_2.5_ concentrations due to vehicular emissions during a given season contributed to the largest spatial variability in their concentrations at the TRAFFIC site, relative to the reference site. Although the PM_2.5_ data were available only for years 2009−2010, annual mean concentrations of PM_2.5_ were approximately four- to five-times higher than the annual mean EU standard. Furthermore, PM_2.5_/PM_10_ ratio based on only 2-year long data suggest PM_2.5_ comprises major proportion PM_10_ concentration. This is of concern given the profound human health relevance of PM_2.5_ exposure. Furthermore, PM_10_ is associated with highly accurate (>92%) and precise (>91%) estimation of ambient PM_2.5_ concentration in all sites except the TRAFFIC site. In contrast, while no clear seasonal variability was seen for NO_2_, the median concentration was particularly elevated near the traffic sites. Median tropospheric ozone concentration remained well-below the EU standard value throughout the 2000−2010 period. During the years 2000−2010, the air quality of Kraków demonstrates an overall city-wide decline in ambient SO_2_ level, which is counterbalanced by the contributions of automobile traffic-related air pollution. 

## Appendix

**Table A1:** Mixed-Effects models of PM_10_, PM_2.5_, SO_2_, NO_2_, and O_3_. IF stands for impact factor.

Predictors		PM_10_			PM_2.5_			SO_2_			NO_2_			O_3_		
		*β*	SE	IF	*β*	SE	IF	*β*	SE	IF	*β*	SE	IF	*β*	SE	IF
Intercept		1.7	0.0	5.6	1.7	0.0	5.4	0.9	0.0	2.6	1.5	0.0	4.3	1.2	0.1	3.2
Year	2000	−0.1	0.0	0.9				0.3	0.0	1.4	0.0	0.0	1.0	−0.3	0.1	0.8
	2001	−0.2	0.0	0.8				0.3	0.0	1.4	0.0	0.0	1.0	−0.2	0.1	0.8
	2002	0.1	0.0	1.1				0.3	0.0	1.3	0.0	0.0	1.0	0.1	0.0	1.1
	2003	0.0	0.0	1.0				0.3	0.0	1.3	0.0	0.0	1.0	0.2	0.0	1.3
	2004	0.0	0.0	1.0				0.2	0.0	1.2	0.0	0.0	1.0	0.1	0.0	1.1
	2005	0.0	0.0	1.0				0.2	0.0	1.2	0.0	0.0	1.0	0.1	0.0	1.1
	2006	0.0	0.0	1.0				0.2	0.0	1.2	0.0	0.0	1.0	0.0	0.0	1.0
	2007	0.0	0.0	1.0				0.1	0.0	1.1	0.0	0.0	1.0	−0.1	0.0	1.0
	2008	−0.1	0.0	1.0				−0.1	0.0	1.0	−0.1	0.0	1.0	0.0	0.0	1.0
	2009	0.0	0.0	1.0	0.0	0.0	1.0	−0.1	0.0	0.9	0.0	0.0	1.0	0.0	0.0	1.0
	2010	0.0		1.0	0.0		1.0	0.0		1.0	0.0		1.0	0.0		1.0
Month	January	0.0	0.0	1.0	0.0	0.0	1.0	0.1	0.0	1.1	0.0	0.0	1.0	0.2	0.0	1.2
	February	0.0	0.0	1.0	0.0	0.0	1.0	0.1	0.0	1.1	0.0	0.0	1.0	0.3	0.0	1.3
	March	−0.1	0.0	1.0	−0.2	0.0	0.8	−0.1	0.0	0.9	0.0	0.0	1.0	0.4	0.0	1.5
	April	−0.1	0.0	0.9	−0.3	0.0	0.7	−0.2	0.0	0.8	0.0	0.0	1.0	0.5	0.0	1.6
	May	−0.3	0.0	0.7	−0.5	0.0	0.6	−0.4	0.0	0.7	−0.1	0.0	0.9	0.5	0.0	1.6
	June	−0.4	0.0	0.7	−0.6	0.0	0.5	−0.4	0.0	0.7	−0.1	0.0	0.9	0.5	0.0	1.6
	July	−0.4	0.0	0.7	−0.6	0.1	0.6	−0.5	0.0	0.6	−0.2	0.0	0.9	0.5	0.0	1.6
	August	−0.3	0.0	0.7	−0.6	0.0	0.5	−0.4	0.0	0.7	−0.1	0.0	0.9	0.4	0.0	1.5
	September	−0.2	0.0	0.8	−0.4	0.0	0.7	−0.4	0.0	0.7	−0.1	0.0	0.9	0.3	0.0	1.4
	October	−0.1	0.0	0.9	−0.2	0.0	0.8	−0.2	0.0	0.8	0.0	0.0	1.0	0.2	0.0	1.2
	November	−0.1	0.0	0.9	−0.1	0.0	0.9	−0.2	0.0	0.9	0.0	0.0	1.0	0.0	0.0	1.0
	December	0.0		1.0	0.0		1.0	0.0		1.0	0.0		1.0	0.0		1.0
Day	Sunday	0.0	0.0	1.0	0.0	0.0	1.0	0.0	0.0	1.0	−0.1	0.0	1.0	0.0	0.0	1.0
	Monday	0.0	0.0	1.0	0.0	0.0	1.0	0.0	0.0	1.0	0.1	0.0	1.1	0.0	0.0	1.0
	Tuesday	0.0	0.0	1.0	0.0	0.0	1.0	0.0	0.0	1.0	0.1	0.0	1.1	−0.1	0.0	0.9
	Wednesday	0.0	0.0	1.0	0.0	0.0	1.0	0.0	0.0	1.0	0.1	0.0	1.1	−0.1	0.0	0.9
	Thursday	0.0	0.0	1.0	0.1	0.0	1.1	0.0	0.0	1.0	0.1	0.0	1.1	−0.1	0.0	0.9
	Friday	0.0	0.0	1.0	0.0	0.0	1.0	0.0	0.0	1.0	0.1	0.0	1.1	−0.1	0.0	0.9
	Saturday	0.0		1.0	0.0		1.0	0.0		1.0	0.0		1.0	0.0		1.0
Site	URBAN	0.0	0.0	1.0	0.0	0.0	1.0	0.1	0.0	1.1	0.0	0.0	1.0	0.1	0.0	1.1
	SUB1	0.0	0.0	1.0				−0.1	0.0	0.9	0.0	0.0	1.0	0.5	0.1	1.7
	TRAFFIC	0.2	0.0	1.2	0.1	0.0	1.1	0.1	0.0	1.1	0.4	0.0	1.4			
	INDU	0.1	0.0	1.1	0.0	0.0	1.0	0.0	0.0	1.0	0.0	0.0	1.0			
	SUB2	−0.1	0.0	1.0				−0.1	0.0	0.9	0.0	0.0	1.0	0.1	0.1	1.1
	CENTRAL	0.0		1.0	0.0		1.0	0.0		1.0	0.0		1.0	0.0		1.0
Temperature	< 4.9	−0.1	0.0	1.0	0.0	0.0	1.0	0.1	0.0	1.1	−0.1	0.0	0.9	−0.1	0.0	0.9
(°C)	4.9−14.6	−0.1	0.0	0.9	0.0	0.0	1.0	0.0	0.0	1.0	−0.1	0.0	1.0	-0.1	0.0	0.9
	≥ 14.6	0.0		1.0	0.0		1.0	0.0		1.0	0.0		1.0	0.0		1.0
Wind speed	< 0.90	0.2	0.0	1.2	0.2	0.0	1.3	0.1	0.0	1.1	0.1	0.0	1.1	−0.1	0.0	0.9
(m/sec)	≥ 0.90	0.0		1.0	0.0		1.0	0.0		1.0	0.0		1.0	0.0		1.0
